# Multimorbidity trajectories and their sex-specific impacts on risk of mortality and re-hospitalisation

**DOI:** 10.1038/s41598-026-41806-7

**Published:** 2026-03-07

**Authors:** Matthew Ennis, Paula L. McClean, Priyank Shukla, Joanna L. Sharman, Ramneek Gupta, Steven Watterson

**Affiliations:** 1 Personalised Medicine Centre, School of Medicine, Ulster University, C-TRIC Building, Altnagelvin Area Hospital, Glenshane Road, Derry~Londonderry, BT47 6SB United Kingdom; 2 Novo Nordisk Research Centre Oxford, The Innovation Building, Roosevelt Dr, Headington, Oxford, OX3 7FZ United Kingdom; 3 Division of Endocrinology and Diabetology, Internal Medicine I, University Hospital of Ulm, Albert-Einstein-Allee 23, 89081 Ulm, Germany

**Keywords:** Multimorbidity, Comorbidity, Multiple long-term conditions, MLTC, Mortality, Hospitalisation, Diseases, Health care, Medical research, Risk factors

## Abstract

The clinical presentation of disease is complicated by multimorbidity, underscoring the need to clarify its effects and identify intervention targets. Using whole UK Biobank hospital inpatient data, we performed the first comprehensive, sex-stratified analysis of age- and morbidity-adjusted mortality and re-hospitalisation outcomes of multimorbidity accrual, both total and specific historical diagnoses, for a range of presenting diagnoses. One-year mortality and re-hospitalisation risks associated with the presenting total accrued multimorbidity of an individual varied markedly with the presenting diagnosis, ranging from no effect to a 1.58-fold increase per diagnosis, highlighting strong context-dependent risks of multimorbidity accrual. Sex disparities in these context-dependent effects were greater for re-hospitalisation than mortality risk. Identification and risk-modelling of non-random trajectories of specific diagnoses revealed especially high-risk multimorbidity (1.16-16.18 fold resultant increases in 1-year mortality/re-hospitalisation) with predictable orders of presentation, suggesting potential points of focused intervention. Notably, a history of diagnosed substance use in males markedly amplified mortality and re-hospitalisation rates, particularly in presentations of anaemias and infections, as did cardiometabolic histories in men presenting with certain digestive diseases.

**Supplementary Information:**

The online version contains supplementary material available at 10.1038/s41598-026-41806-7.

## Introduction

Multimorbidity, defined as the simultaneous diagnosis of two or more long term conditions, is common, but not well understood. A recent meta-analysis estimated global prevalence of multimorbidity at 37.2%^1^. Multimorbidity appears to be growing. Quadrupling in the prevalence of patients with five or more conditions from 2000 to 2014 has been reported^2^. Incidence has been linked to socioeconomic deprivation with patients in the most deprived decile in Scotland experiencing 4.6% more morbidities than patients in the least deprived decile, across all ages^3^.

The clinical impact of multimorbidity is significant and it has been identified as an international health priority^4,5^. Multi-morbid patients account for 58% of primary care patients and 78% of primary care consultations^6^. Prescriptions accumulate with increasing multimorbidity, making multimorbidity also a driver of polypharmacy^7,8^. Increasing polypharmacy has been reported in a Scottish population with the number of patients receiving 5 or more and 10 or more drugs growing from 11.4% and 1.7% in 1995 to 21.1% and 5.8%, respectively, in 2010^9^. Reducing the burden of multimorbidity is an important step in achieving the Sustainable Development Goal of reducing premature mortality from non-communicable diseases by one-third by 2030^1^.

The UK Biobank (UKBB) comprises data on 502,386 participants from England, Scotland and Wales aged between 40 and 69 at recruitment^10^. Access to clinical and molecular data make it well suited to studies of disease coincidence. Several studies have been conducted to explore how diagnoses statistically cluster in patient records of the UKBB. Hierarchical clustering and association rule mining have been applied to 36 chronic conditions, identifying 3 clusters which highlight the importance of diabetes, hypertension and asthma in multimorbidity^11^. A clustering of over 400 diseases based on risk factors identified 24 clusters and found strong sex-dependent associations of disease risk with BMI^12^. Another study identified 5 clusters in UKBB by applying Charlson’s broad disease classification and multiple correspondence analysis^13^. However, little work has been done on how diagnoses dynamically accrue over time, as patients’ age and multimorbidity increases, and few links have previously been made with how a patient’s risk of outcomes such as mortality or re-hospitalisation change as diseases accrue.

A study utilising the Danish National Patient Registry to evaluate the influence of disease history on sepsis mortality revealed that prior trajectories of multimorbidity, many of which start from alcohol abuse, diabetes, and cardiovascular disease, increase risk of death^14^. This approach has illustrated the potential for the use of temporal diagnosis trajectories to identify patient subgroups at high risk of mortality/hospitalisation. However, there has not been an attempt to compare mortality risk associated with multimorbidity patterns across a broad spectrum of common diseases nor to identify index diseases within multimorbid patients which confer highest mortality risk.

Here, we explore the dynamic accrual of multimorbidity of participants from the UKBB and identify diagnosis presentations where accrued multimorbidity results in severe risk of re-hospitalisation and death. We assess the effects of total multimorbidity on mortality and re-hospitalisation risk of commonly presenting diagnoses in secondary care. We then identify specific high-risk trajectories of diagnoses which have a predictable order of presentation, suggesting potential subgroups for targeted intervention. In each case, we explore sexual dimorphism in patterns and outcomes. Insights identified here may be incorporated into care pathways to ensure interventions may be targeted to patients with the greatest need, improving the efficiency and efficacy of multimorbidity treatment.

## Results

### Characterisation of diagnosis accrual across the UK biobank

**Fig. 1:**
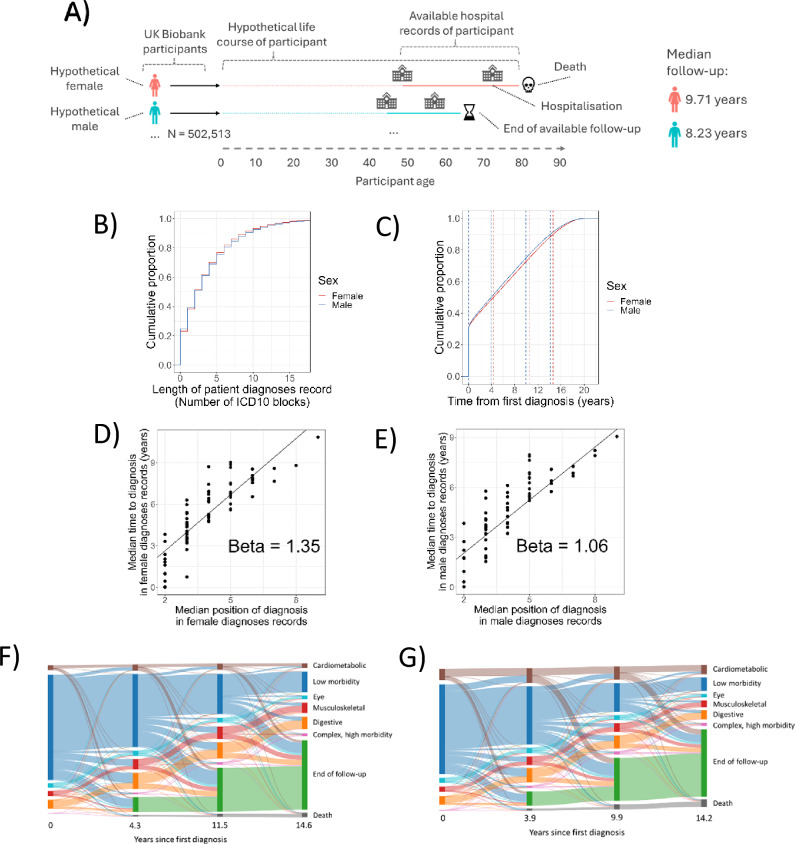
ICD10 diagnosis sequence and timing for females and males determined from UK Biobank secondary care data. (**A**) Illustrative structure and hypothetical examples of the input hospitalisation data derived from the UK Biobank. (**B**) The cumulative proportion of females (red) (N = 273,388) and males (blue) (N = 229,125) who have been diagnosed with a number of morbidities, or fewer, by the end of the study period. (**C**) The cumulative proportion of diagnoses from the time of first diagnosis for females (red) and males (blue). The time scale is divided into 4 quantiles (25%, 50%, 75%, 90%) (dashed lines). (**D**) Median time to diagnosis versus median position of diagnosis in patient diagnoses records for each ICD10 block for females and (**E**) for males. (**F**) Multimorbid transitions of all females with more than 1 ICD10 block within their records (N = 168,276) as they move between/within clusters across the quantiles shown in Figure 1C. (G) Multimorbid transitions of all males with more than 1 ICD10 block within their records (N = 139,353) between/within the clusters across the quantiles shown in Figure 1B.

We obtained ICD10-coded diagnoses and dates of diagnoses from inpatient hospital admission records available for UKBB participants (Figure 1A) and excluded ICD10 codes describing procedures/interactions with healthcare that do not describe disease conditions. ICD10 data was measured at the level of ICD10 blocks (See ‘Methods’). The median follow-up time from the time of first available recorded hospitalisation was 9.71 years for females and 8.23 years for males (Figure 1A). Most patients in the UK biobank have multiple diagnoses, with 61.6% of 273,388 female and 60.8% of 229,125 male patients diagnosed with 2 or more diagnoses (Figure 1B). As diagnoses accrue, the order and timing can vary between patients. We calculated the duration from the patients’ first diagnosis to each subsequent diagnosis. From the first diagnosis, 50% of total diagnoses occur after 3.9 years for males and 4.3 years for females (Figure 1C). We also calculated the position of each diagnosis in the order of diagnoses. Median and quartiles were calculated (see Supplementary Figure 1) and for each ICD10 block we compared the median position in diagnosis order to median time to diagnosis from first recorded diagnosis for females (Figure 1D) and males (Figure 1E).

The relationship between position in patient diagnosis records and the time-to-diagnosis is broadly linear in both females (Figure 1D) and males (Figure 1E). We see there is variation around the trendline, indicating that there are certain patient groups who accrue more, or less, multimorbidity over a given period than expected (Supplementary Tables 1-2). The trend line rises more steeply for females than males, suggesting that females accrue diagnoses more slowly than males (Figure 1D-E).

We subsequently focused further analysis on those females and males who exhibited multimorbidity (more than 1 diagnosed ICD10 block). We defined 6 clusters from the ICD10 block diagnosis combinations of multimorbid females (N = 168,276) and males (N = 139,353) and observed how participants transition between clusters over their available follow-up period (Figure 1F-G; Supplementary Figure 2; Supplementary Tables 3-5). Clusters were derived from k-means clustering of dimensionally-reduced diagnosis data encompassing both females and males. Based on the diagnosis enrichment within clusters (Supplementary Tables 4-5), clusters were labelled as ‘Complex, high morbidity’, ‘Low morbidity’, ‘Digestive’, ‘Eye’, ‘Cardiometabolic’, and ‘Musculoskeletal’. Interestingly, 25.2% of males at first presentation (T = 0) fit a multimorbidity cluster pattern other than ‘Low morbidity’ (Supplementary Table 3). In contrast, the same figure for females was 18.1% with the greatest difference observed in the frequency of the Cardiometabolic cluster at presentation (9.3% of males versus 3.7% of females; Figure 1F-G; Supplementary Table 3) indicating that multimorbidity, particularly cardiometabolic multimorbidity, is more advanced in males than females at first presentation in secondary care.

Mortality is lower for female than male participants. A greater proportion of female participants stayed in the low morbidity cluster over available follow-up and a greater proportion of female participants transitioned into the Digestive or Musculoskeletal clusters. In comparison, a greater proportion of male participants transitioned into the Cardiometabolic cluster. Complex, high morbidity was rarely found at first presentation and took the longest of all patterns to develop with most female and males entering the ‘Complex, high morbidity’ cluster between 11.5-14.6 and 9.9-14.2 years after first diagnosis, respectively (Figure 1F-G; Supplementary Table 3).

Whole-cohort clustering provides a low dimensional representation of the dynamics of the dataset but do not clearly provide specific points of possible intervention, limiting the translational value of the analysis. Consequently, we sought to investigate specific diagnosis presentations.

### Sensitivity of mortality and re-hospitalisation risk to total multimorbidity in common secondary care presentations

**Fig. 2:**
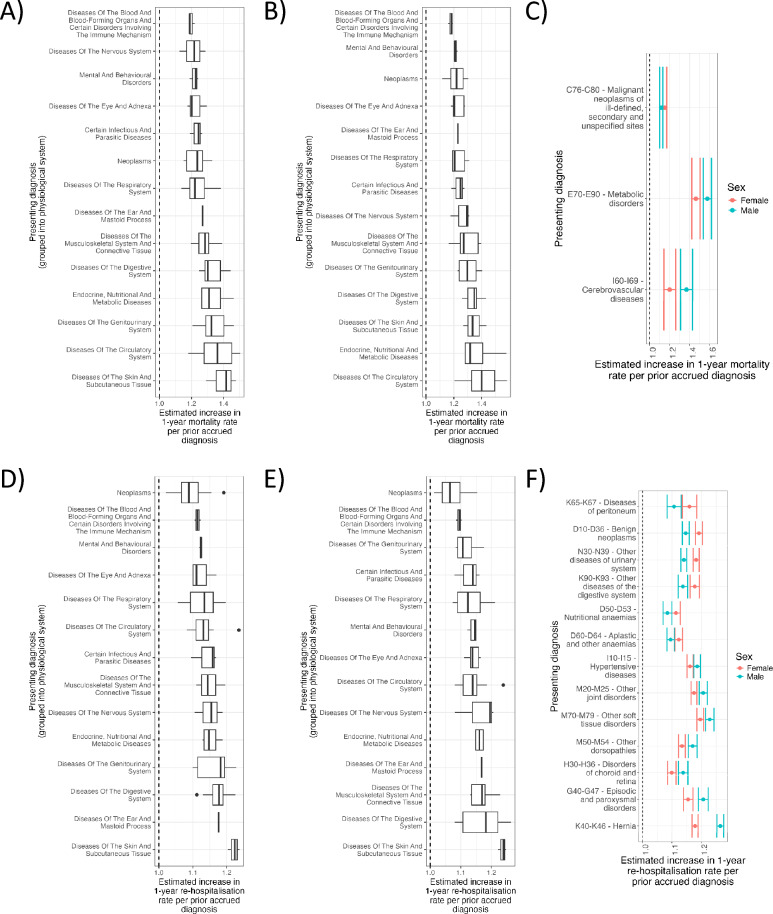
The 1-year mortality and re-hospitalisation risk of increasingly multimorbid presentations is dependent on the presenting diagnosis and sex. Presenting diagnoses are grouped by their respective ICD10 chapter indicating affected physiological system. (**A**) Female age-adjusted estimates of increase in 1-year mortality risk per prior diagnosis for specific presenting diagnoses. (**B**) Male age-adjusted estimates of increase in 1-year mortality risk per prior diagnosis for specific presenting diagnoses. (**C**) Combined female and male 1-year mortality risk estimates per prior diagnosis for specific presenting diagnoses ordered by difference in effect size between females and males. Only those with non-overlapping confidence intervals between sexes are shown. (**D**) Female age-adjusted estimates of increase in 1-year re-hospitalisation risk per prior diagnosis for specific presenting diagnoses. (**E**) Male age-adjusted estimates of increase in 1-year re-hospitalisation risk per prior diagnosis for specific presenting diagnoses. (**F**) Combined female and male 1-year re-hospitalisation risk estimates per prior diagnosis for specific presenting diagnoses ordered by difference in effect size between females and males. Only those with non-overlapping confidence intervals between sexes are shown.

We assessed how the effect of accumulated total multimorbidity (the total number of accrued diagnoses) on 1-year mortality and re-hospitalisation risk varied with the presenting diagnosis in females and males separately. For each patient diagnosed with a given diagnosis (the ‘presenting diagnosis’) we calculated the number of pre-existing and co-presenting diagnoses the patient had accrued by the time of the presenting diagnosis under consideration. We subsequently used Accelerated Failure Time (AFT) models^26^ to estimate the effect of total accrued multimorbidity on mortality and re-hospitalisation risk 1-year post-presenting diagnosis. AFT models were adjusted for age by the time of the presenting diagnosis (Figure 2 and Supplementary Tables 6-9).

We found that the effect of total presenting multimorbidity on 1-year mortality and re-hospitalisation risk was highly dependent on the presenting diagnosis in both females and males (Figure 2A-B and 2D-E), indicating significant context-dependent effects of accrued multimorbidity on risk. The median fold increase in 1-year mortality rate with each prior accrued diagnosis across all diagnosis presentations was 1.27x and 1.28x in females and males, respectively, and varied from 1.12x to 1.50x in females and from 1.12x to 1.58x in males (Supplementary Figure 3A-B and Supplementary Tables 6-7). The median fold increase in 1-year re-hospitalisation rates with each prior accrued diagnosis across all diagnosis presentations was 1.14x in both females and males and varied from no effect to 1.24x in females and from no effect to 1.26x in males (Supplementary Figure 3C-D and Supplementary Tables 8 and 9).

Broadly, we observed that 1-year mortality risk was most sensitive to total multimorbidity in presentations of circulatory and skin disorders in both females and males. Mortality risk was specifically most sensitive to total multimorbidity within presentations of Other forms of heart disease (I30-I52) (effect Females = 1.50x per prior diagnosis, adj.p = 2.820 x 10^-98^; effect Males = 1.57x per prior diagnosis, adj.p = 1.484 x 10^-193^) (Figure 2A-B and Supplementary Tables 6-7), indicating that the multimorbidity burden of patients presenting with non-ischaemic forms of heart disease is a major predictor of 1-year mortality, in both females and males.

In contrast, mortality rates 1-year following presentations of blood and mental disorders were the least sensitive to presenting multimorbidity. The 1-year mortality risk specifically least sensitive to multimorbidity was Malignant neoplasms of ill-defined, other secondary and unspecified sites (C76-C80) (effect Females = 1.15x per prior diagnosis, adj.p = 2.469 x 10^-62^; effect Males = 1.12x per prior diagnosis, adj.p = 1.578 x 10^-52^) (Figure 2A-B and Supplementary Tables 6 and 7), indicating that the multimorbid burden of patients presenting with secondary malignancies is a comparatively more minor predictor of 1-year mortality.

Interestingly, we observed sex-dependent effects in the sensitivity of mortality risk to presenting multimorbidity (Figure 2C). Notably, Male 1-year mortality risk was significantly more sensitive to total accrued multimorbidity in the presentation of Cerebrovascular diseases (I60-I69) (effect Males = 1.37x; effect Females = 1.20x), indicating multimorbidity burden is a greater predictor of 1-year mortality in males presenting with stroke than in females.

One-year re-hospitalisation risk for presentations of skin or digestive orders were the most sensitive to total multimorbidity in both females and males, while presentations of cancers or blood disorders were least sensitive (Figure 2D-E). Future re-hospitalisation risk was particularly sensitive to total multimorbidity for both females and males presenting with Other disorders of the skin and subcutaneous tissue (L80-L99) (effect Females = 1.24x per prior diagnosis, adj.p = 4.387 x 10^-288^; effect Males = 1.24x per prior diagnosis, adj.p = 2.900 x 10^-280^). Interestingly, we found the 1-year re-hospitalisation risk in females and males presenting with Malignant neoplasms of digestive organs (C15-C26) to be insignificantly related to total accrued multimorbidity, indicating no differences in 1-year healthcare utilisation between patients with low and high multimorbidity (Female adj.p = 0.055; male adj.p = 0.217) (Figure 2D-E and Supplementary Tables 8 and 9).

In contrast with mortality, sex disparities in re-hospitalisation risk associated with increasing total multimorbidity were more pervasive, suggesting that increasingly multimorbid females and males often experience differing rates of healthcare utilisation. The most female-skewed diagnosis in terms of sensitivity of 1-year re-hospitalisation risk to prior multimorbidity was Diseases of peritoneum (K65-K67) (effect Females = 1.16x; effect Males = 1.11x) while the most male-skewed diagnosis was Hernia (K40-K46) (effect Males = 1.26x; effect Females = 1.18x) (Figure 2F and Supplementary Tables 8 and 9).

These results demonstrate that the effects of multimorbidity on mortality and re-hospitalisation risk are highly contextual, suggesting interventions targeting multimorbidity in secondary care should be prioritised to specific presenting patient groups, such as those presenting with circulatory or skin disorders. These contextual effects are further dependent on sex, particularly for re-hospitalisation risk.

### High-risk multimorbidity with predictable orders of presentation

We next investigated whether we could identify specific forms of multimorbidity which resulted in an especially high 1-year mortality and/or re-hospitalisation risk, independently of the total presenting multimorbidity. We limited our initial analysis to pairs of diagnoses which were significantly coincident in participants, excluding diagnosis pairs with no evidence of a significant relationship. We further limited our analysis to pairs of diagnoses which exhibited predictable orders of presentation, thereby indicating potential points for intervention. We refer to combinations of related diagnoses with predictable orders of presentation as ‘diagnosis trajectories’. These diagnosis trajectories represent frequently observed subsequences of complete sequences of diagnoses from patient records (See Supplementary Tables 10 and 11 and Supplementary Figures 4-9). For each of these identified trajectories, we defined the ‘presenting’ diagnosis as the final occurring diagnosis in the trajectory while the diagnosis occurring before the presenting was defined as the ‘historical’ diagnosis. We subsequently used AFT models to identify historical diagnoses associated with mortality and re-hospitalisation 1-year following each associated presenting diagnosis (Supplementary Tables 12-15). Effects on both mortality and hospitalisation were adjusted for the age and total prior number of diagnoses of participants at the time of the presenting diagnosis, including the number of co-presenting diagnoses. A full description of the construction of diagnosis trajectories is provided in ‘Methods’.

The coincidence rates of diagnosis pairs were highly correlated between females and males (r = 0.9, p < 0.001), with males exhibiting on average higher rates of coincidence of diagnoses (Supplementary figure 4D). We observed several notable diagnosis pairs which deviated significantly from this broad trend including higher coincidence rates of Disorders of thyroid gland (E00-E07) with Hypertensive diseases (I10-I15), Metabolic disorders (E70-E90), and Arthrosis (M15-M19). Conversely, men had substantially greatly coincidence rates of cardiometabolic diseases and secondary malignancies of digestive cancers (Supplementary Figure 4D). Amongst diagnosis trajectories common to both sexes (39.7% of female and 40.2% of male trajectories), rates of development (in years to last diagnosis since first diagnosis) were also highly correlated (r = 0.95, p < 0.001) (Supplementary Tables 10 and 11), indicating almost no variation in trajectory development between females and males when the order of diagnosis development is consistent. Additionally, only 3 significant diagnosis pairs exhibited significant diagnosis orders that were differing between females and males, indicating that the ordering of diagnoses between females and males is relatively consistent (Supplementary Tables 10 and 11).

We note that the effects of age on 1-year mortality and hospitalisation differs substantially between trajectories (Supplementary Tables 12-15). However, this variation is generally linked to the presenting diagnosis rather than with differences in historical diagnoses, indicating that risk of outcomes is differentially modulated by age across presenting diagnoses rather than trajectories per se.

### Mortality and re-hospitalisation rates of diagnosis trajectories

We observed that cancer histories (‘C00-D49 – Neoplasms’) had large effects on the 1-year mortality and re-hospitalisation risk for a wide number of presenting diagnoses, with most exhibiting increased risk (Supplementary Figure 10). We therefore distinguished between those diagnosis histories that were cancers and those that were not. Broadly, the large effect of historical cancer diagnoses on 1-year mortality and re-hospitalisation risk were independent of the presenting diagnosis. This is also in accordance with the observed minor effects of total multimorbidity on 1-year outcomes in cancer presentations, indicating that the 1-year mortality and re-hospitalisation risks of new-onset cancers are more independent of multimorbidity. These high-risk historical cancer diagnoses, including secondary malignances and digestive cancers, frequently presented in hospitalisations due to various genitourinary, digestive, respiratory, and infectious diseases (Supplementary Figure 10 and Supplementary Tables 12-15).

### Non-cancer multimorbidity resulting in a high risk of mortality and hospitalisation

**Fig. 3:**
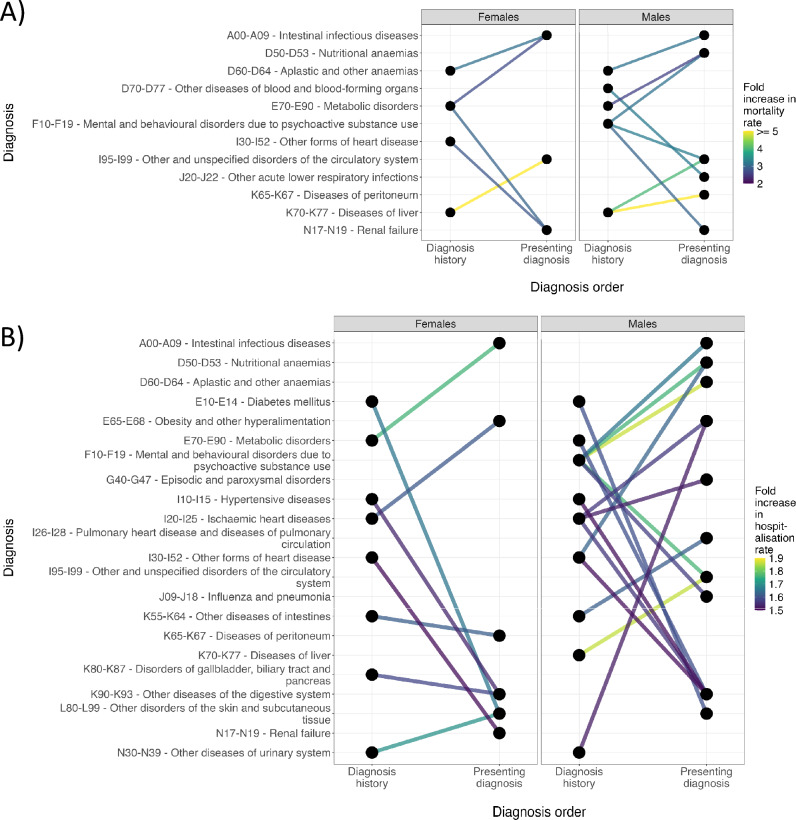
Diagnosis trajectories identify high-risk non-cancer multimorbidity with typical orders of presentation. We visualised single non-cancer diagnosis histories significantly associated with 1-year mortality and hospitalisation risk independently of age and total prior multimorbidity for females and males. Only diagnosis histories with greater than 2.5 or 1.5 fold increases in 1-year mortality or re-hospitalisation risk are shown. (**A**) Female and male single non-cancer diagnosis histories significantly associated with increased 1-year mortality risk for a given presenting diagnoses. (**B**) Female and male single non-cancer diagnosis histories significantly associated with increased 1-year hospitalisation risk for a given presenting diagnosis.

We visualised non-cancer diagnosis histories significantly associated with increased 1-year mortality and re-hospitalisation for given presenting diagnoses to identify high-risk forms of multimorbidity beyond those involving pre-existing cancer (Figure 3 and Supplementary Tables 12-15). In each instance, we report the numbers of individuals who presented with both the presenting diagnosis and the diagnosis history, either as a historical or a co-presenting diagnosis.

We identified 6 instances of non-cancer diagnosis histories significantly associated with increased 1-year mortality rates in females, 5 of which were associated with >2.5-fold increase in 1-year mortality rates (Figures 3A). The greatest 1-year mortality risk observed in females was a history of Diseases of liver (K70-K77) in patients presenting with Other and unspecified disorders of the circulatory system (I95-I99) where patients with this history (N = 303) had a 6.67x higher rate of 1-year mortality than those without (Adj.p = 3.14 x 10^-4^). Other forms of non-cancer multimorbidity with high 1-year mortality risk in females included females presenting with Renal failure (N17-N19) along with either a history of Other forms of heart disease (I30-I52) (N = 1749) or Metabolic disorders (E70-E90) (N = 3023) or females presenting with Intestinal infectious diseases (A00-A09) along with a history of Aplastic and other anaemias (D60-D64) (N = 874) or Metabolic disorders (E70-E90) (N = 2145) (Figure 3A and Supplementary Table 12).

For males, we identified 17 instances of non-cancer diagnosis histories significantly associated with increased 1-year mortality rates, 8 of which were associated with 2.5-fold increase in 1-year mortality rates. (Figure 3A). The greatest risk observed in males was within patients presenting with Diseases of peritoneum (K65-K67) along with a history of Diseases of liver (K70-K77) (N = 227) where 1-year mortality risk was 16.18x greater (Adj.p = 1.80 x 10^-7^) than those without this history (Figure 3A). As within females, a history of Aplastic and other anaemias (D60-D64) in males presenting with Intestinal infectious diseases (N = 752) was also associated with significant 1-year mortality. Interestingly, we uniquely found in males that a history of diagnosed Mental and behavioural disorders due to psychoactive substance use (F10-F19) was associated with 1-year mortality in several presenting diagnoses (1.822-3.254x greater 1-year mortality) including Nutritional anaemias (D50-D53) (N = 582), Other and unspecified disorders of the circulatory system (I95-I99) (N = 873), and Renal failure (N17-N19) (N = 1380) (Figure 3A and Supplementary Table 13). This indicates that substance use-associated multimorbidity in males is a significant predictor of mortality.

For 1-year risk of re-hospitalisation, we identified 22 and 25 instances of non-cancer diagnosis histories significantly associated with elevated risk, in females and males, respectively (Figure 3B). The greatest effect on 1-year re-hospitalisation risk in females was observed for patients presenting with Intestinal infectious diseases (A00-A09) where those with a history of Metabolic disorders (E70-E90) (N = 1568) had 1.761x higher rates of 1-year re-hospitalisation than those without this history (adj.p = 1.61 x 10^-11^) (Figure 3B). Other forms of multimorbidity with a high risk of 1-year re-hospitalisation included females presenting with Other disorders of the skin and subcutaneous tissue (L80-L99) along with a history of either Diabetes mellitus (E10-E14) (1.66x greater 1-year re-hospitalisation rate) (N = 744) or Other diseases of urinary system (N30-N39) (1.70x greater 1-year re-hospitalisation rate) (N = 1249) (Figure 3B and Supplementary Tables 14-15).

Within males, we observed the greatest increase in 1-year re-hospitalisation risk with those presenting with Aplastic and other anaemias (D60-D64) along with a history of Mental and behavioural disorders due to psychoactive substance use (F10-F19) (1.866x increase in 1-year re-hospitalisation rate, adj.p = 1.09 x 10^-9^) (Figure 3B). As with mortality risk, a history of Mental and behavioural disorders due to psychoactive substance use (F10-F19) also commonly increased 1-year re-hospitalisation rates in males (Figure 3B). Broadly, historical diagnoses of substance use disorders significantly increased 1-year re-hospitalisation risk in males presenting with various anaemias or infections. Another unique observation within males was the significant increase in 1-year re-hospitalisation rates associated with historical cardiometabolic diagnoses in males presenting with Other diseases of the digestive system (K90-K93), of which histories of Metabolic disorders (E70-E90) and Ischaemic heart diseases (I20-I25) exhibited the greatest associated risks (Figure 3B and Supplementary Table 15). This indicates that multimorbidity involving cardiometabolic and certain digestive disorders in males results in significant healthcare utilisation.

In totality, we identified specific forms of multimorbidity which both associate with significant risk of mortality and/or re-hospitalisation and which have a predictable order of development, suggesting potential points of early intervention. Broadly, we found that historical diagnoses of substance use in males substantially elevated mortality and re-hospitalisation risk, particularly within presentations of anaemias or infections, as did historical diagnoses of cardiometabolic diseases in male presentations of certain digestive disorders.

### Additive and interactive effects of multi-diagnosis histories on mortality and hospitalisation

We further assessed whether a more detailed assessment considering multiple historical diagnoses could indicate multimorbid subgroups with even greater risks of 1-year mortality and/or re-hospitalisation. To do so, we constructed diagnosis trajectories of lengths greater than 2 and tested the associated 1-year mortality and re-hospitalisation risk of each historical diagnosis simultaneously in multivariate AFT models. We further considered whether multi-diagnosis histories increased risk additively or multiplicatively through interaction effects.

We found no evidence of significant positive interaction effects of multi-diagnosis histories for either mortality or hospitalisation, indicating that specific multi-diagnosis histories do not increase risk multiplicatively (Supplementary Figure 11 and Supplementary Tables 16-19). Significant risk-increasing multi-diagnosis histories were predominantly ‘cancer + cancer’ and ‘cancer + non-cancer’ histories and represented patient groups of sizes between 32 to 660 (Supplementary Figure 11 and Supplementary Tables 16-19).

Amongst female multimorbid histories involving at least 1 non-cancer diagnosis, we broadly observed that simultaneous histories of cancer with either cardiovascular or musculoskeletal diseases lead to substantial 1-year risk of mortality and re-hospitalisation (Supplementary Figure 11A and 11C and Supplementary Tables 16 and 18). The largest of these effects in females was for 1-year mortality in females presenting with Renal failure (N17-N19) along with either historical diagnoses of both Hypertensive diseases (I10-I15) and Malignant neoplasms of ill-defined, other secondary and unspecified sites (C76-C80) (combined effect = 70.86 fold greater 1-year mortality) (N with both at presentation = 377) or both Malignant neoplasm of breast (C50-C50) and Other forms of heart disease (I30-I52) (combined effect = 40.83 fold greater 1-year mortality) (N with both at presentation = 114) (Supplementary Figure 11A and Supplementary Table 16). This indicates that multimorbid females with renal failure, cancer and cardiovascular diseases represent a complex group with substantial mortality risk.

Amongst male multi-diagnosis histories involving at least 1 non-cancer diagnosis, 1-year mortality and re-hospitalisation risk-increasing multimorbidity predominantly involved historical diagnoses of Malignant neoplasms of ill-defined, other secondary and unspecified sites (C76-C80) and Mental and behavioural disorders due to psychoactive substance use (F10-F19), with these 2 histories together being the most frequently observed combination (Supplementary Figure 11B and 11D and Supplementary Tables 17 and 19). This indicates that the mortality and re-hospitalisation risks of cancers and substance use in males are largely independent, highlighting a complex form of multimorbidity in males associated with substantially elevated risk.

## Discussion

As far as we are aware, this is the first hypothesis-free study of mortality and re-hospitalisation outcomes of multimorbidity accrual to use data covering a broad range of common diagnoses as they present to clinicians in secondary care. Few studies have investigated trajectories of multimorbidity and fewer still have focused on trajectories of time-ordered diagnosis sequences and their associated outcomes^15^. Whilst some studies have looked at differences in disease progression patterns in men and women^16^, we are not aware of any which considered sex differences in outcomes. In the present study, we observed several differences between females and males in the sensitivity of mortality and hospitalisation risk to both total prior multimorbidity burden (Figure 2) and specific prior histories of diagnoses (Figure 3).

We show that clinicians should consider both the total multimorbidity burden and specific histories of diagnoses when assessing a patient’s mortality and hospitalisation risk. The total diagnosis burden appears to substantially associate with risk, such as for mortality risk in patients presenting with cerebrovascular diseases, particularly in males (Figure 2C). We provide presentation-specific estimates of the effect of total prior disease burden coded in ICD10 on 1-year mortality and hospitalisation risk which could be readily implemented in secondary care. In particular, we highlight the significant effects on 1-year mortality and re-hospitalisation of presenting total multimorbidity in patients presenting with circulatory or skin disorders. Further research providing a greater understanding of the underlying correlates of risk with increasing total multimorbidity burden, which is likely a proxy for other factors such as frailty^17^, may allow the replacement of total diagnosis burden for more interpretable predictors.

The predictive power of trajectories of time-ordered diagnoses sequences has been investigated for mortality of sepsis patients^14^. Patients were identified in a Danish cohort using electronic healthcare records using the ICD10 code Other sepsis (A41) and the authors found complex prior trajectories involving alcohol abuse, cardiovascular, diabetes, anaemias, and/or cancers to be associated with increased sepsis mortality. We corroborate that patients presenting with Other bacterial diseases (A30-A49) with prior histories of cancer, anaemias, or substance abuse have significantly higher mortality risk than those without such prior histories but do not find that prior histories of cardiometabolic disease to be significant. We also found substance abuse to only carry mortality risk within males. We further extend this prior work beyond sepsis and identify specific multimorbid subgroups with significant 1-year mortality and hospitalisation risk of patients presenting with anaemias, metabolic disorders, intestinal infections, renal failure, pneumonia/influenza, and more.

We observed historical diagnoses of cancer, particularly secondary malignancies and digestive cancers, to almost universally present the greatest risk, independent of total multimorbidity burden, for both 1-year mortality and re-hospitalisation risk. Interestingly, we also observed the 1-year mortality and re-hospitalisation risk in patients presenting with secondary malignancies and digestive cancers to be particularly insensitive to total presenting multimorbidity burden. In contrast, the mortality and re-hospitalisation risk of patients presenting with breast, skin, and male genital organ cancers appears to be more sensitive to total multimorbidity, highlighting cancers where risk is significantly increased by multimorbidity. A recent study investigated the effect of multimorbidity on overall survival in females in South Africa with breast cancer and found newly-diagnosed patients with multimorbidity to have poorer overall 3-year survival than non-multimorbid patients^18^. Our results agree with this finding, as we found 1-year mortality risk in newly diagnosed breast cancer patients to exhibit the greatest sensitivity to prior total multimorbidity amongst cancer patient groups in females.

Triage of patients within secondary care is currently employed using various scoring systems such as Acute Physiology and Chronic Health Evaluation, Simplified Acute Physiology Score, or Mortality Probability Model^19^. Although some of these scoring systems do use a select number of pre-existing conditions for assessing patients, the number utilised is small and does not make use of the wealth of diagnosis history data that is presently available in patient Electronic Heathcare Records (EHRs)^19^. Given the increasing digitalisation of healthcare, future scoring systems for triage in secondary care may benefit from integration with and consideration of EHR secondary care diagnosis data. Other digital health technologies may also improve the management of multimorbidity, including wearable devices, telehealth platforms, and the Internet of Things, the latter of which has been proposed for improving surgical care^20,21^. Our identification of specific high-risk multimorbid subgroups based on diagnosis histories provides simple qualitative rules which may aid in improving triage of the growing population of multimorbid patients.

## Online methods

### Formatting and filtering of patient diagnosis records

We extracted the data field ‘41270: Diagnoses – ICD10’ for participants in the UK Biobank, containing diagnoses from available inpatient admissions records coded in ICD10 and converted the ICD10 codes to groupings of ICD10 blocks and ICD10 chapters. As an example, the ICD10 codes ‘I20 - Angina pectoris’ and ‘I25 - Chronic ischaemic heart disease’ were both converted to the ICD10 block ‘I20-I25 - Ischaemic heart diseases’ and to the ICD10 chapter ‘I00-I99 – Diseases of the circulatory system’. We choose the level of ICD10 blocks as the primary level of analysis in order to analyse a broad set of disease diagnoses while maintaining both a manageable number of diagnosis codes and sufficient sample sizes within diagnosis groups. Diagnoses included both the primary diagnosis which was causal for admission as well as secondary diagnoses. We extracted the data field ‘41280: Date of first in-patient diagnosis - ICD10’ from the inpatient admissions records, containing the date of occurrence of each ICD10 diagnosis. Each participant’s diagnostic history was described as a vector of ICD10 blocks ordered by date of diagnosis. Only the earliest recorded instance of each block was used for analysis, while subsequent duplicates were removed. We sex-stratified the data using the field ’31: Sex’ and filtered for ICD10 blocks with more than 1% prevalence within each sex. Blocks not describing diseases but procedures/interactions with healthcare were also removed, corresponding to ICD10 blocks beginning with letters O, P, Q, R, S, T, U, V, W, X, Y, Z. For each participant, the order of diagnosis was determined and the period to each subsequent diagnosis was calculated from the first diagnosis in days. Diagnoses that occurred on the same date were placed in an arbitrary order of diagnosis. Though suboptimal, in our study this approach only affects the calculation of the median diagnosis order of diseases which is an aggregate measure across the entire population for which we would expect the effect to be negligible.

### Identifying patient diagnosis groups with more or less multimorbidity than expected at diagnosis

To identify patient groups who had accrued more or less multimorbidity than expected at diagnosis, we calculated the median order of each diagnosis in patient records and median time to each diagnosis in days since first available hospitalisation within patient records. After plotting median order against median time, we calculated the line of best fit and the orthonormal basis vectors parallel and perpendicular to the line of best fit. Projecting the data points on to the basis vectors yielded coordinates along these directions. We then calculated the standard deviation and identified the blocks lying outside one and two standard deviations of the mean as exhibiting more, or less, multimorbidity than expected.

### Multimorbidity clustering

We clustered all participants with more than one ICD10 block diagnosis to explore the transitions between clusters over time. The period of record availability was divided into three periods in which diagnoses accumulated using the 25%, 50%, 75%, and 90% quantile thresholds of the distribution of time of diagnosis in days from first recorded diagnosis. Diagnosis combinations were calculated at the start of each period and at the end of the last period. Participants who had died or who no longer had any additional follow-up within a period were removed from the subsequent period. Time of death of participants was calculated relative to the date of their first available hospitalisation using the date of death (obtained from data-field ‘40000: Date of death). Available follow-up for participants who had not died during the study period was set to the most recent overall available date in the female and male diagnosis data.

Clustering was subsequently performed on the data obtained by merging female and male diagnosis combinations and merging across all time points. The tabulated combined data was represented as a diagnosis matrix with rows representing individuals and columns representing diagnoses. Dimensionality reduction was first performed using Multiple Correspondence Analysis (MCA) from the ‘FactoMineR’ R package. The number of components to retain was determined with an elbow plot (Supplementary Figure 2A). The resulting space of coordinates was used in k-means clustering using base R. Optimal cluster parameters were selected using elbow and Calinski-Harabasz^22^ plots (Supplementary Figure 2B-C). Calinski-Harabasz scores were calculated using the ‘fpc’ R package. The movement of individuals between clusters across the four timepoints was visualised as a Sankey diagram using SankeyMATIC. (https://www.sankeymatic.com/)

### Identifying diagnoses sharing trajectories

We measured coincidence between pairs of diagnoses using the Jaccard index, which expresses the proportion of overlap between two groups. If ${N}_{A\cap B}$ denotes the number of participants with both diagnoses A and B in their patient records and ${N}_{A\cup B}$ denotes the number of participants with diagnosis A or diagnosis B in their patient records, the Jaccard Index is defined to be ${N}_{A\cap B}/{N}_{A\cup B}$. Jaccard values were calculated using the ‘jaccard’ package in R. ICD10 chapters were clustered using complete hierarchical clustering on the mean Jaccard values across blocks and blocks were further hierarchically clustered within chapters. Clustering and associated heatmaps were generated with the ‘Heatmap()’ function of the ‘ComplexHeatmap’ package in R.

### Building diagnosis sequence trajectories

In order to build trajectories of significant sequences of diagnoses from the ordered vectors of patient diagnosis records, we adapted an approach developed elsewhere^23^. We identified pairs of diagnoses in multimorbid females and males that were significantly coincident using a bootstrapping test from the ‘jaccard’ R package^24^. For each diagnosis pair, A and B, we calculated the frequency of occurrences where the time to diagnosis of A is less than B, greater than B and equal to B. We subsequently performed double one-tailed binomial tests of whether A or B more frequently occurs earlier, each time including the cases where A and B are equal in time of diagnosis in the comparison group. From the diagnosis pairs with both a significant Jaccard coefficient and a significant ordering, we assembled longer trajectories incrementally based on overlapping diagnoses between trajectories of length 2. Binomial tests were repeated for each longer trajectory, identifying new diagnoses that significantly occurred before or after the longer trajectory in the population of participants with all the diagnoses in the longer trajectory. As an example, from 2 trajectories of length 2, A -> B and A ->C, we tested whether B tended to come before or after C in the population of individuals diagnosed with A, B, and C. Trajectories that affected less than 20 individuals were ignored. This process was repeated until no further trajectories were identified. P-values for all statistical tests were Bonferroni-Holm adjusted^25^.

### Calculating the 1-year mortality and re-hospitalisation risk of prior multimorbidity

We constructed baseline Accelerated Failure Time (AFT) models^26^ which estimated the effect of total prior multimorbidity burden on the 1-year mortality and re-hospitalisation rates of every analysed disease diagnosis in multimorbid females and males at the point of diagnosis. We controlled for age within AFT models by calculating the age of each participant at the time of diagnosis for each respective diagnosis. Age at diagnosis was approximated for each diagnosis patient group from their age at assessment (data field ‘21003: - Age when attended assessment centre’) adjusted by the number of days between their assessment centre visit (obtained from data-field ’53: Date of attending assessment centre’) and the date at which they were diagnosed plus 182.625 (midpoint of a year accounting for leap years). We further calculated and included as a covariate in AFT models the total number of prior diagnoses accrued by the time of diagnosis of the presenting diagnosis for each patient. We subtracted 1 from this calculated number in order to prevent double-counting of the diagnosis burden of the presenting diagnosis. Mortality events were derived from dates of death (obtained from data-field ‘40000: Date of death) while hospitalisation events were derived from unfiltered hospital diagnoses data including ICD10 blocks below 1% prevalence and codes not describing diseases. Mortality models were censored for available follow up in the UK Biobank while re-hospitalisation models were censored for both available follow up and death. In total, this resulted in 144 and 140 baseline AFT models in females and males, respectively. Each model thus provided estimates of the age-relative effect of total prior accrued multimorbidity on the 1-year mortality and hospitalisation risk for 72 and 70 presenting diagnoses in females and males, respectively.

To estimate the effect of prior trajectories of specific diagnoses, we included additional covariates in baseline AFT models indicating whether participants had a history of or co-presented with a specific diagnosis at the time of the presenting diagnosis. We assessed those specific histories of diagnoses which were identified as prior diagnoses of the presenting diagnosis from significant diagnosis sequence trajectories. In this way, we thus assessed the risk of those orderings of diagnoses which were most common. For trajectories of length greater than 2, we further included interaction terms in AFT models between prior diagnosis history covariates in order to test for multiplicative effects on risk of having more than one of the tested diagnosis histories. The effect sizes of significant multi-diagnosis histories were calculated by summing coefficients. The calculated prior total multimorbidity of each patient was adjusted to prevent both double-counting of the presenting diagnosis and the tested diagnosis histories. For example, if a trajectory of length 3 was being tested, an individual with both of the diagnosis histories would have 2 subtracted from their total prior multimorbidity covariate to account for these histories and a further 1 subtracted to account for the presenting diagnosis.

In total, this resulted in the construction of 3568 and 3520 AFT models in females and males, respectively. Each model thus provided estimates of the effect of having accrued specific combinations of diagnoses by the time of the presenting diagnosis on 1-year mortality and hospitalisation rates, independent of age and total prior multimorbidity. P-values for all statistical tests were Bonferroni-Holm adjusted^25^. AFT models were constructed using the ‘survreg()’ function from the ‘survival’ R package. The ‘dist’ parameter within ‘survreg()’ function was set to ‘lognormal’.

### Code/scripts

Scripts reproducing analysis are available from https://github.com/m4ennis/UKB-trajectories.

## Conclusions

We performed a sex-stratified analysis of UK Biobank secondary care and for the first time comprehensively assessed outcomes of multimorbidity accrual, both for total and specific trajectories of diagnoses. We identified significant and novel associations between prior multimorbidity accrual, both for total burden and specific histories of diagnoses, and 1-year risk of mortality and re-hospitalisation for a range of commonly presenting diagnoses. We show that the sensitivity of 1-year mortality and hospitalisation risk to total multimorbidity burden is highly dependent on the presenting diagnosis and is modulated by sex, particularly 1-year re-hospitalisation risk. We identified specific high-risk non-cancer trajectories of diagnoses that occur in a predictable order of presentation, suggesting the possibility of early intervention. We believe that the identified associations could be used to improve triage of complex patients in secondary care and inform further research on high-risk forms of multimorbidity, to identify preventative strategies and design appropriate interventions to improve patient outcomes.

## Supplementary Information

Supplementary Information 1.

Supplementary Information 2.

## Data Availability

UK Biobank data is available by application at https://www.ukbiobank.ac.uk/use-our-data/apply-for-access/.
