# Supplementary material for: Multimorbidity trajectories and their sex-specific impacts on risk of mortality and re-hospitalisation
Source: Sci Rep. 2026 Mar 7;16:12490. doi: 10.1038/s41598-026-41806-7 (PMC13087196; doi:10.1038/s41598-026-41806-7)
Supplement: Supplementary file 1 — Supplementary Information 1. [file 41598_2026_41806_MOESM1_ESM.pptx]

## Slide 1
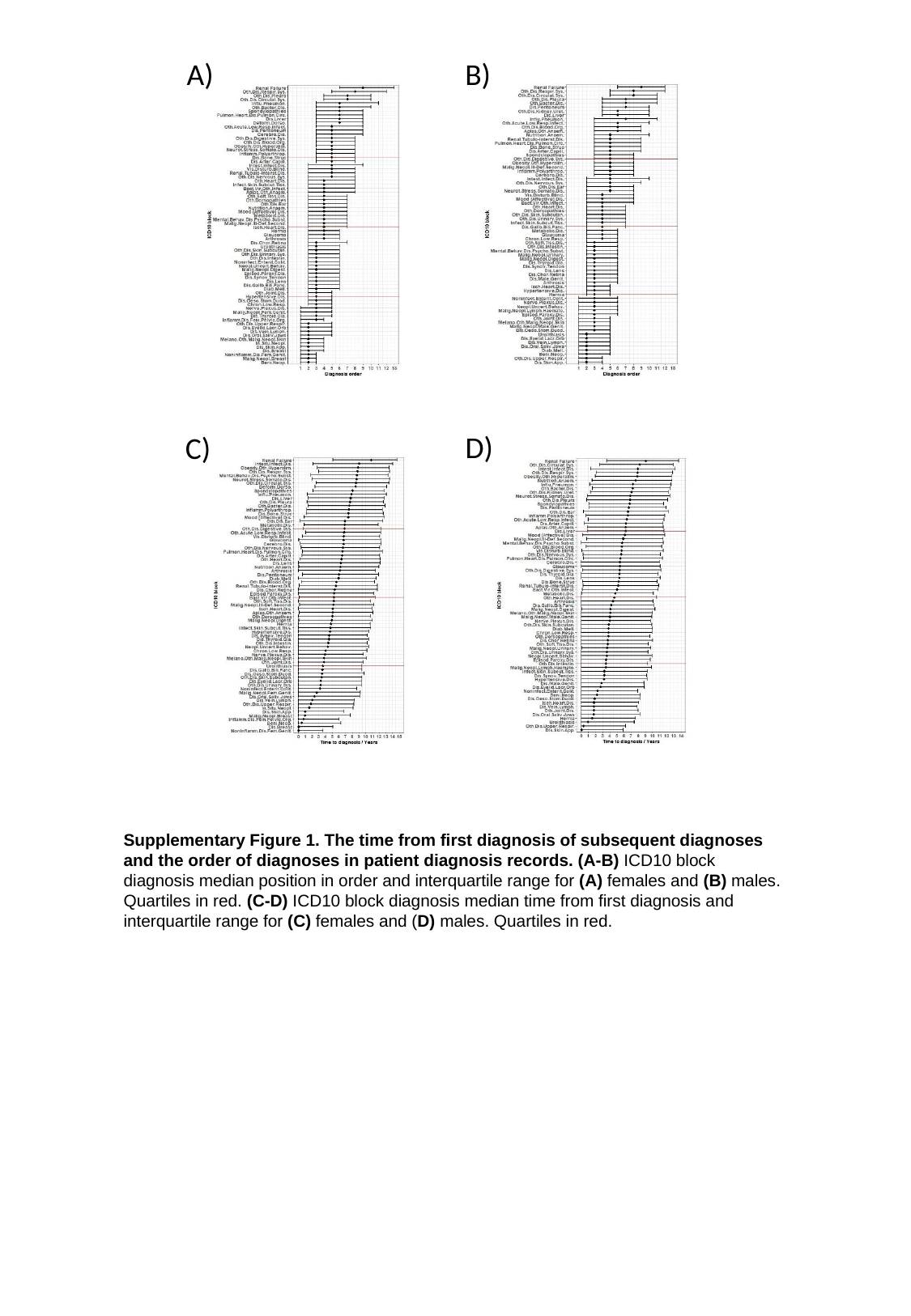

A)
B)
D)
C)
Supplementary Figure 1. The time from first diagnosis of subsequent diagnoses and the order of diagnoses in patient diagnosis records. (A-B) ICD10 block diagnosis median position in order and interquartile range for (A) females and (B) males. Quartiles in red. (C-D) ICD10 block diagnosis median time from first diagnosis and interquartile range for (C) females and (D) males. Quartiles in red.

## Slide 2
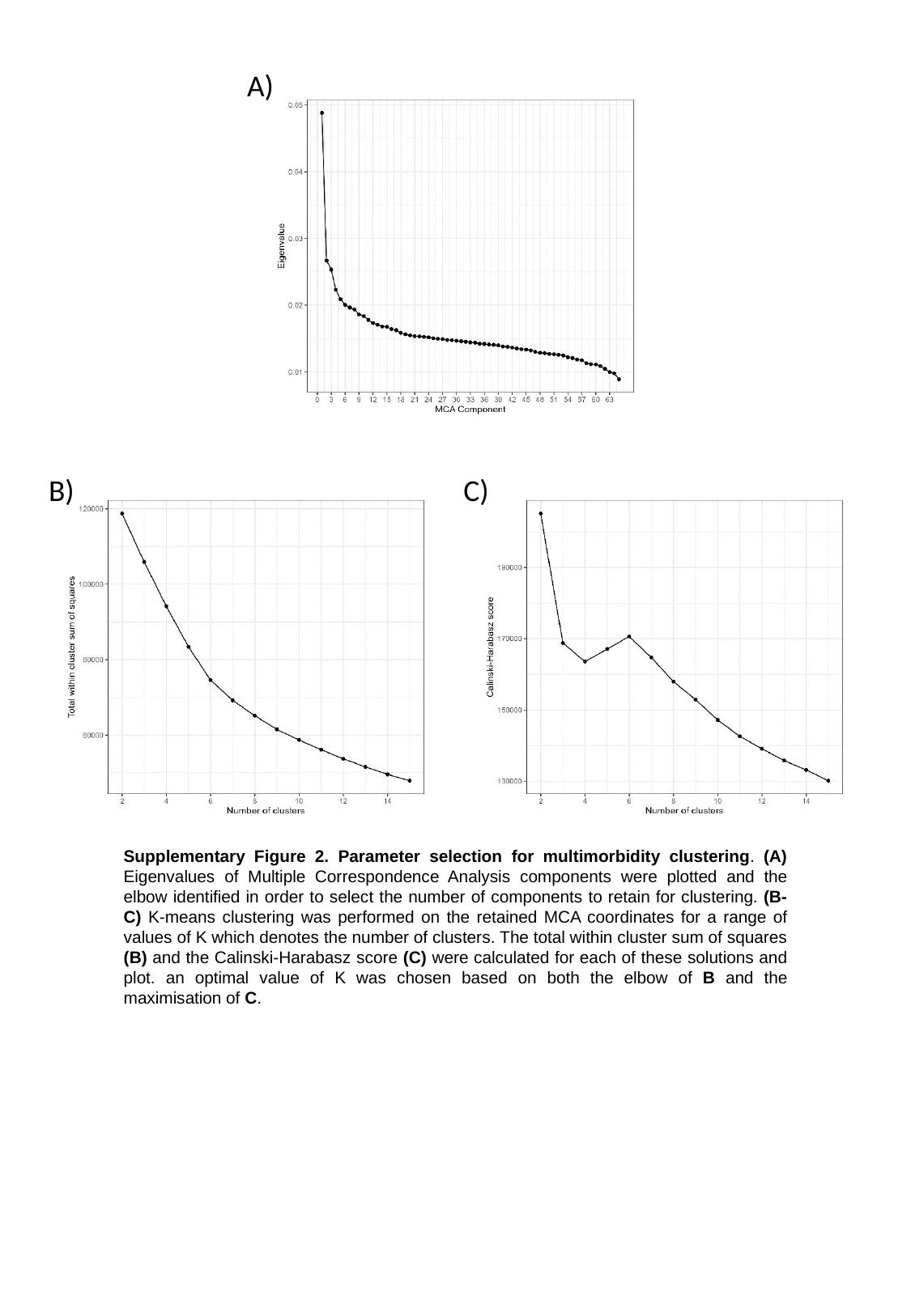

A)
B)
C)
Supplementary Figure 2. Parameter selection for multimorbidity clustering. (A) Eigenvalues of Multiple Correspondence Analysis components were plotted and the elbow identified in order to select the number of components to retain for clustering. (B-C) K-means clustering was performed on the retained MCA coordinates for a range of values of K which denotes the number of clusters. The total within cluster sum of squares (B) and the Calinski-Harabasz score (C) were calculated for each of these solutions and plot. an optimal value of K was chosen based on both the elbow of B and the maximisation of C.

## Slide 3
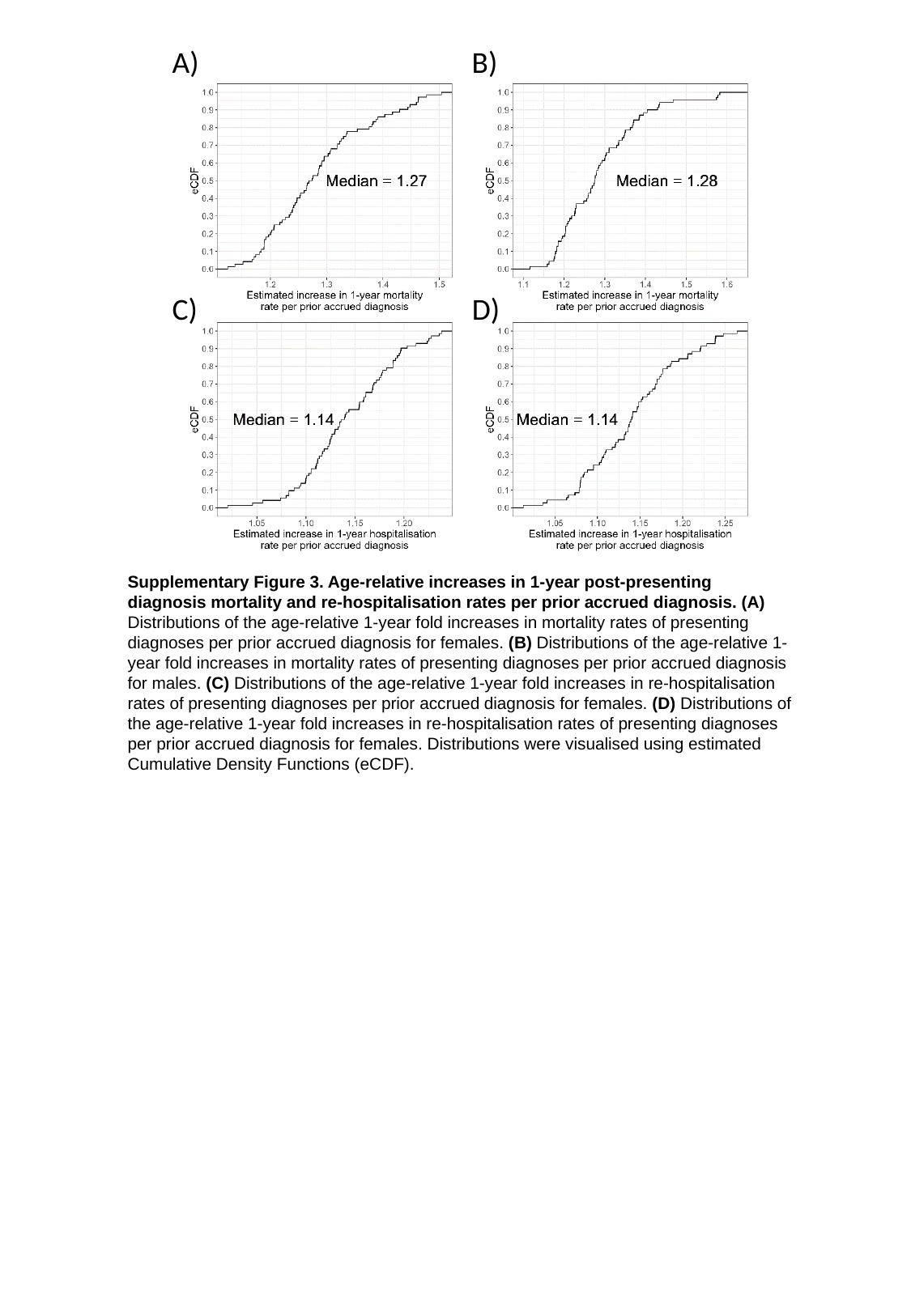

A)
B)
C)
D)
Supplementary Figure 3. Age-relative increases in 1-year post-presenting diagnosis mortality and re-hospitalisation rates per prior accrued diagnosis. (A) Distributions of the age-relative 1-year fold increases in mortality rates of presenting diagnoses per prior accrued diagnosis for females. (B) Distributions of the age-relative 1-year fold increases in mortality rates of presenting diagnoses per prior accrued diagnosis for males. (C) Distributions of the age-relative 1-year fold increases in re-hospitalisation rates of presenting diagnoses per prior accrued diagnosis for females. (D) Distributions of the age-relative 1-year fold increases in re-hospitalisation rates of presenting diagnoses per prior accrued diagnosis for females. Distributions were visualised using estimated Cumulative Density Functions (eCDF).

## Slide 4
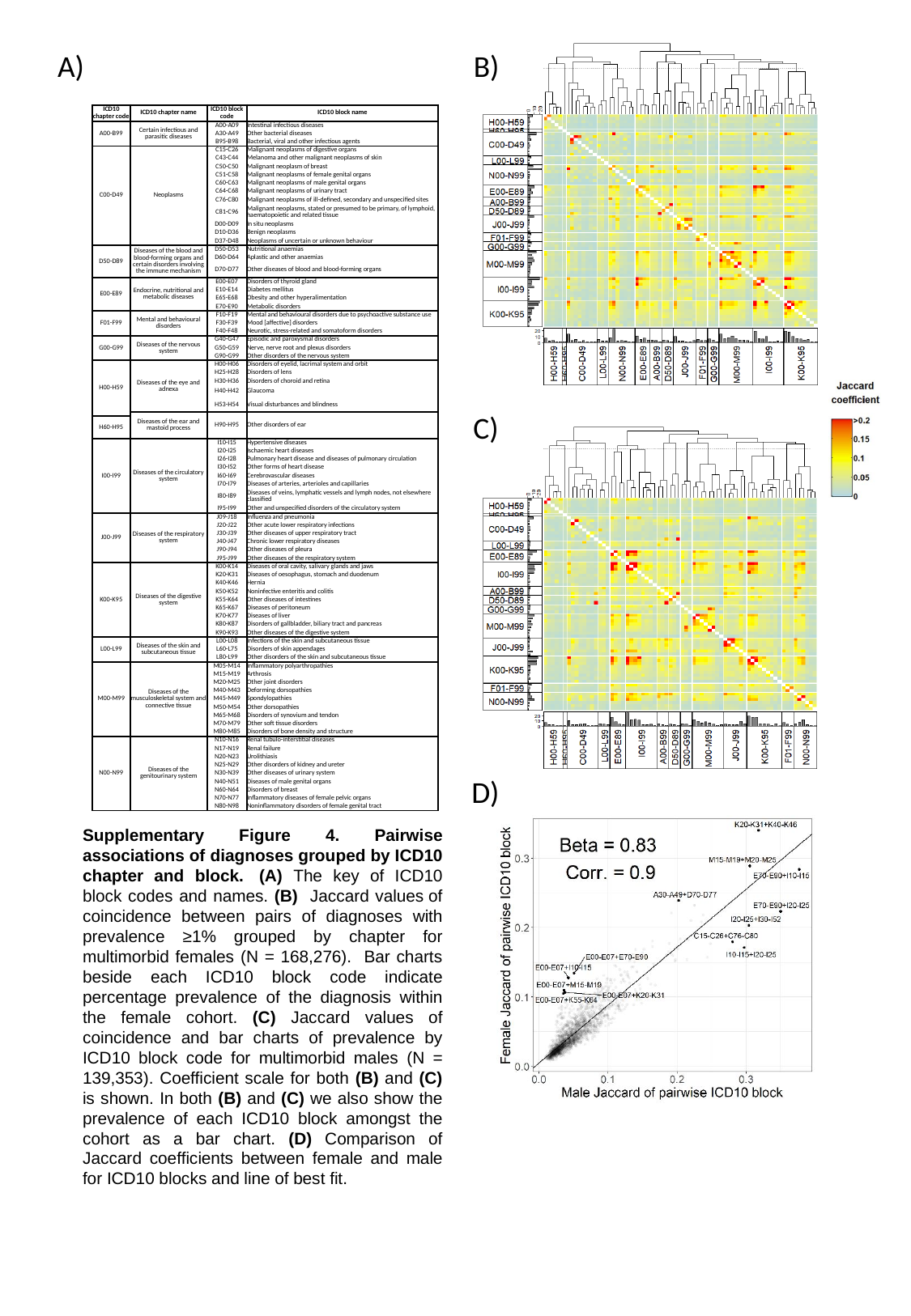

A)
B)
| ICD10 chapter code | ICD10 chapter name | ICD10 block code | ICD10 block name |
| --- | --- | --- | --- |
| A00-B99 | Certain infectious and parasitic diseases | A00-A09 | Intestinal infectious diseases |
| | | A30-A49 | Other bacterial diseases |
| | | B95-B98 | Bacterial, viral and other infectious agents |
| C00-D49 | Neoplasms | C15-C26 | Malignant neoplasms of digestive organs |
| | | C43-C44 | Melanoma and other malignant neoplasms of skin |
| | | C50-C50 | Malignant neoplasm of breast |
| | | C51-C58 | Malignant neoplasms of female genital organs |
| | | C60-C63 | Malignant neoplasms of male genital organs |
| | | C64-C68 | Malignant neoplasms of urinary tract |
| | | C76-C80 | Malignant neoplasms of ill-defined, secondary and unspecified sites |
| | | C81-C96 | Malignant neoplasms, stated or presumed to be primary, of lymphoid, haematopoietic and related tissue |
| | | D00-D09 | In situ neoplasms |
| | | D10-D36 | Benign neoplasms |
| | | D37-D48 | Neoplasms of uncertain or unknown behaviour |
| D50-D89 | Diseases of the blood and blood-forming organs and certain disorders involving the immune mechanism | D50-D53 | Nutritional anaemias |
| | | D60-D64 | Aplastic and other anaemias |
| | | D70-D77 | Other diseases of blood and blood-forming organs |
| E00-E89 | Endocrine, nutritional and metabolic diseases | E00-E07 | Disorders of thyroid gland |
| | | E10-E14 | Diabetes mellitus |
| | | E65-E68 | Obesity and other hyperalimentation |
| | | E70-E90 | Metabolic disorders |
| F01-F99 | Mental and behavioural disorders | F10-F19 | Mental and behavioural disorders due to psychoactive substance use |
| | | F30-F39 | Mood [affective] disorders |
| | | F40-F48 | Neurotic, stress-related and somatoform disorders |
| G00-G99 | Diseases of the nervous system | G40-G47 | Episodic and paroxysmal disorders |
| | | G50-G59 | Nerve, nerve root and plexus disorders |
| | | G90-G99 | Other disorders of the nervous system |
| H00-H59 | Diseases of the eye and adnexa | H00-H06 | Disorders of eyelid, lacrimal system and orbit |
| | | H25-H28 | Disorders of lens |
| | | H30-H36 | Disorders of choroid and retina |
| | | H40-H42 | Glaucoma |
| | | H53-H54 | Visual disturbances and blindness |
| | Diseases of the ear and mastoid process | H90-H95 | Other disorders of ear |
| H60-H95 | | | |
| I00-I99 | Diseases of the circulatory system | I10-I15 | Hypertensive diseases |
| | | I20-I25 | Ischaemic heart diseases |
| | | I26-I28 | Pulmonary heart disease and diseases of pulmonary circulation |
| | | I30-I52 | Other forms of heart disease |
| | | I60-I69 | Cerebrovascular diseases |
| | | I70-I79 | Diseases of arteries, arterioles and capillaries |
| | | I80-I89 | Diseases of veins, lymphatic vessels and lymph nodes, not elsewhere classified |
| | | I95-I99 | Other and unspecified disorders of the circulatory system |
| J00-J99 | Diseases of the respiratory system | J09-J18 | Influenza and pneumonia |
| | | J20-J22 | Other acute lower respiratory infections |
| | | J30-J39 | Other diseases of upper respiratory tract |
| | | J40-J47 | Chronic lower respiratory diseases |
| | | J90-J94 | Other diseases of pleura |
| | | J95-J99 | Other diseases of the respiratory system |
| K00-K95 | Diseases of the digestive system | K00-K14 | Diseases of oral cavity, salivary glands and jaws |
| | | K20-K31 | Diseases of oesophagus, stomach and duodenum |
| | | K40-K46 | Hernia |
| | | K50-K52 | Noninfective enteritis and colitis |
| | | K55-K64 | Other diseases of intestines |
| | | K65-K67 | Diseases of peritoneum |
| | | K70-K77 | Diseases of liver |
| | | K80-K87 | Disorders of gallbladder, biliary tract and pancreas |
| | | K90-K93 | Other diseases of the digestive system |
| L00-L99 | Diseases of the skin and subcutaneous tissue | L00-L08 | Infections of the skin and subcutaneous tissue |
| | | L60-L75 | Disorders of skin appendages |
| | | L80-L99 | Other disorders of the skin and subcutaneous tissue |
| M00-M99 | Diseases of the musculoskeletal system and connective tissue | M05-M14 | Inflammatory polyarthropathies |
| | | M15-M19 | Arthrosis |
| | | M20-M25 | Other joint disorders |
| | | M40-M43 | Deforming dorsopathies |
| | | M45-M49 | Spondylopathies |
| | | M50-M54 | Other dorsopathies |
| | | M65-M68 | Disorders of synovium and tendon |
| | | M70-M79 | Other soft tissue disorders |
| | | M80-M85 | Disorders of bone density and structure |
| N00-N99 | Diseases of the genitourinary system | N10-N16 | Renal tubulo-interstitial diseases |
| | | N17-N19 | Renal failure |
| | | N20-N23 | Urolithiasis |
| | | N25-N29 | Other disorders of kidney and ureter |
| | | N30-N39 | Other diseases of urinary system |
| | | N40-N51 | Diseases of male genital organs |
| | | N60-N64 | Disorders of breast |
| | | N70-N77 | Inflammatory diseases of female pelvic organs |
| | | N80-N98 | Noninflammatory disorders of female genital tract |
C)
D)
Supplementary Figure 4. Pairwise associations of diagnoses grouped by ICD10 chapter and block.  (A) The key of ICD10 block codes and names. (B) Jaccard values of coincidence between pairs of diagnoses with prevalence ≥1% grouped by chapter for multimorbid females (N = 168,276). Bar charts beside each ICD10 block code indicate percentage prevalence of the diagnosis within the female cohort. (C) Jaccard values of coincidence and bar charts of prevalence by ICD10 block code for multimorbid males (N = 139,353). Coefficient scale for both (B) and (C) is shown. In both (B) and (C) we also show the prevalence of each ICD10 block amongst the cohort as a bar chart. (D) Comparison of Jaccard coefficients between female and male for ICD10 blocks and line of best fit.

## Slide 5
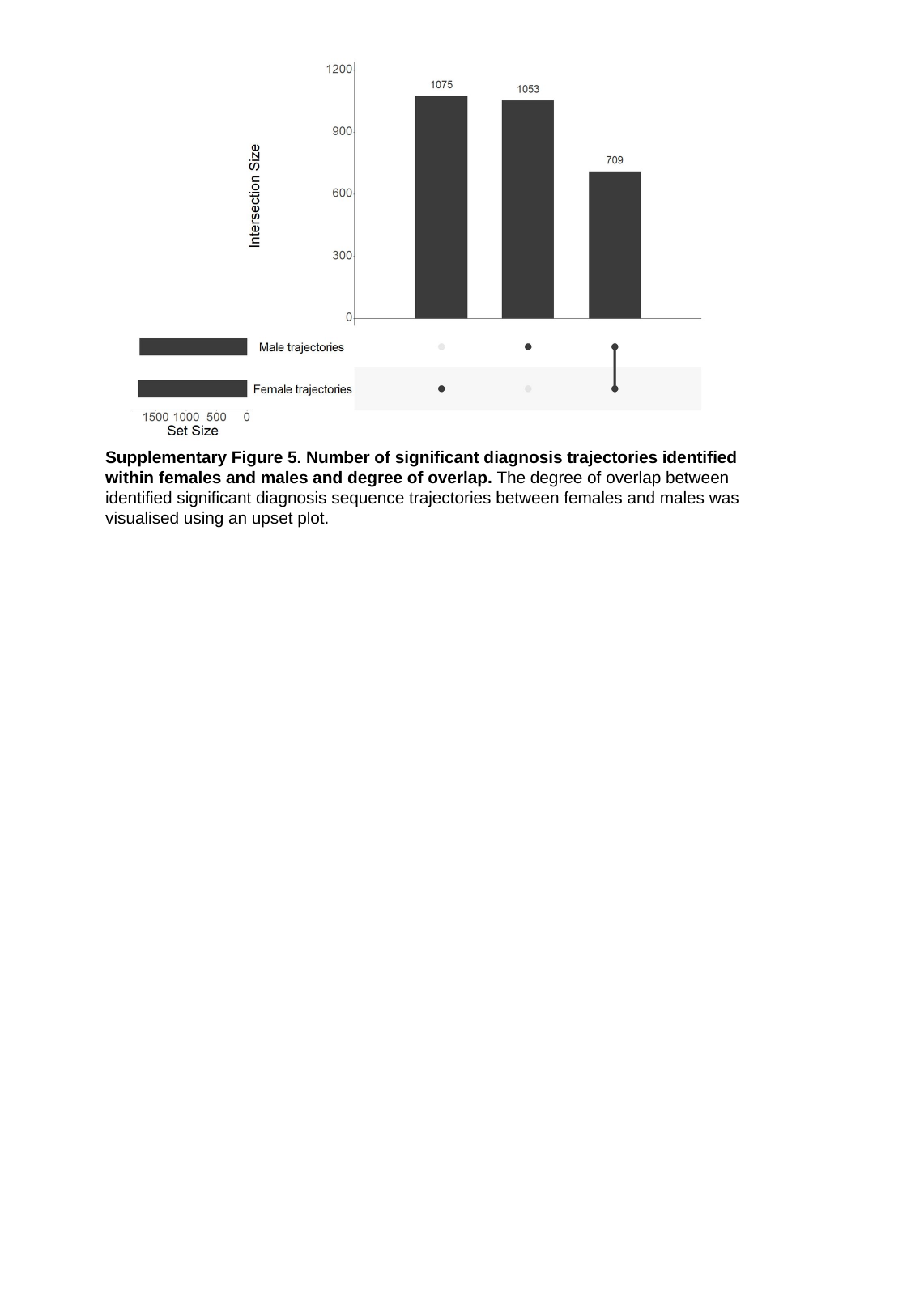

Supplementary Figure 5. Number of significant diagnosis trajectories identified within females and males and degree of overlap. The degree of overlap between identified significant diagnosis sequence trajectories between females and males was visualised using an upset plot.

## Slide 6
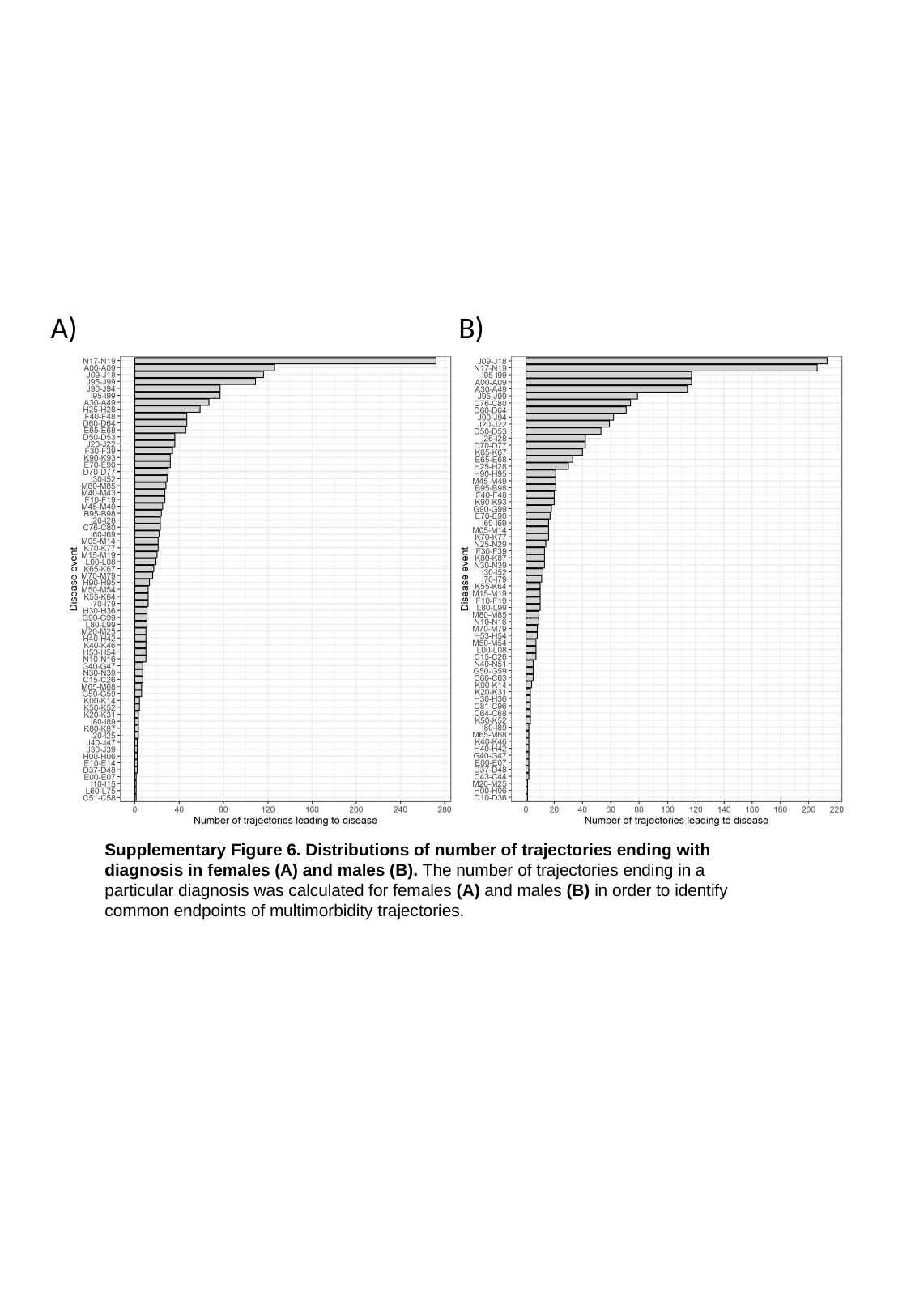

A)
B)
Supplementary Figure 6. Distributions of number of trajectories ending with diagnosis in females (A) and males (B). The number of trajectories ending in a particular diagnosis was calculated for females (A) and males (B) in order to identify common endpoints of multimorbidity trajectories.

## Slide 7
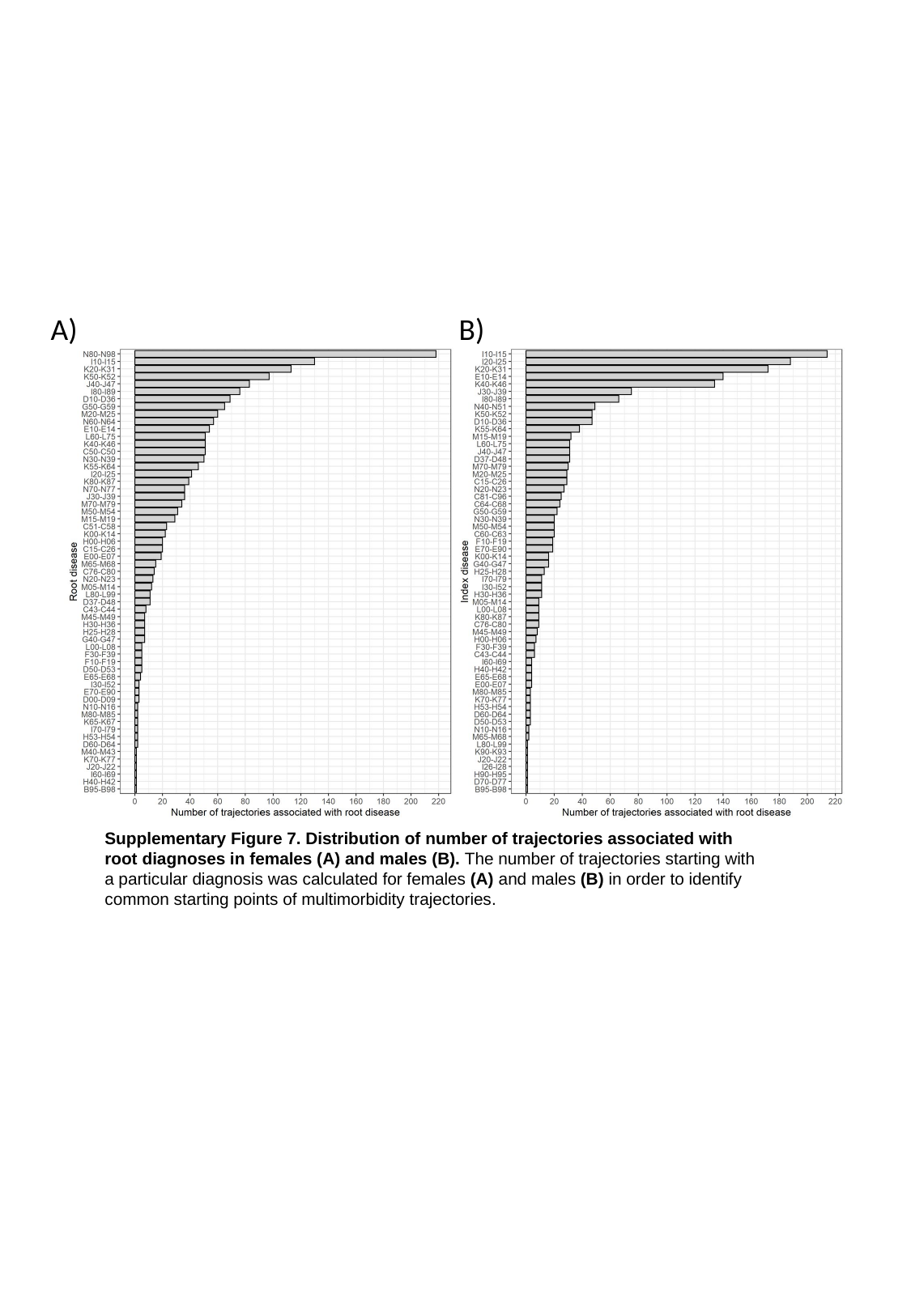

A)
B)
Supplementary Figure 7. Distribution of number of trajectories associated with root diagnoses in females (A) and males (B). The number of trajectories starting with a particular diagnosis was calculated for females (A) and males (B) in order to identify common starting points of multimorbidity trajectories.

## Slide 8
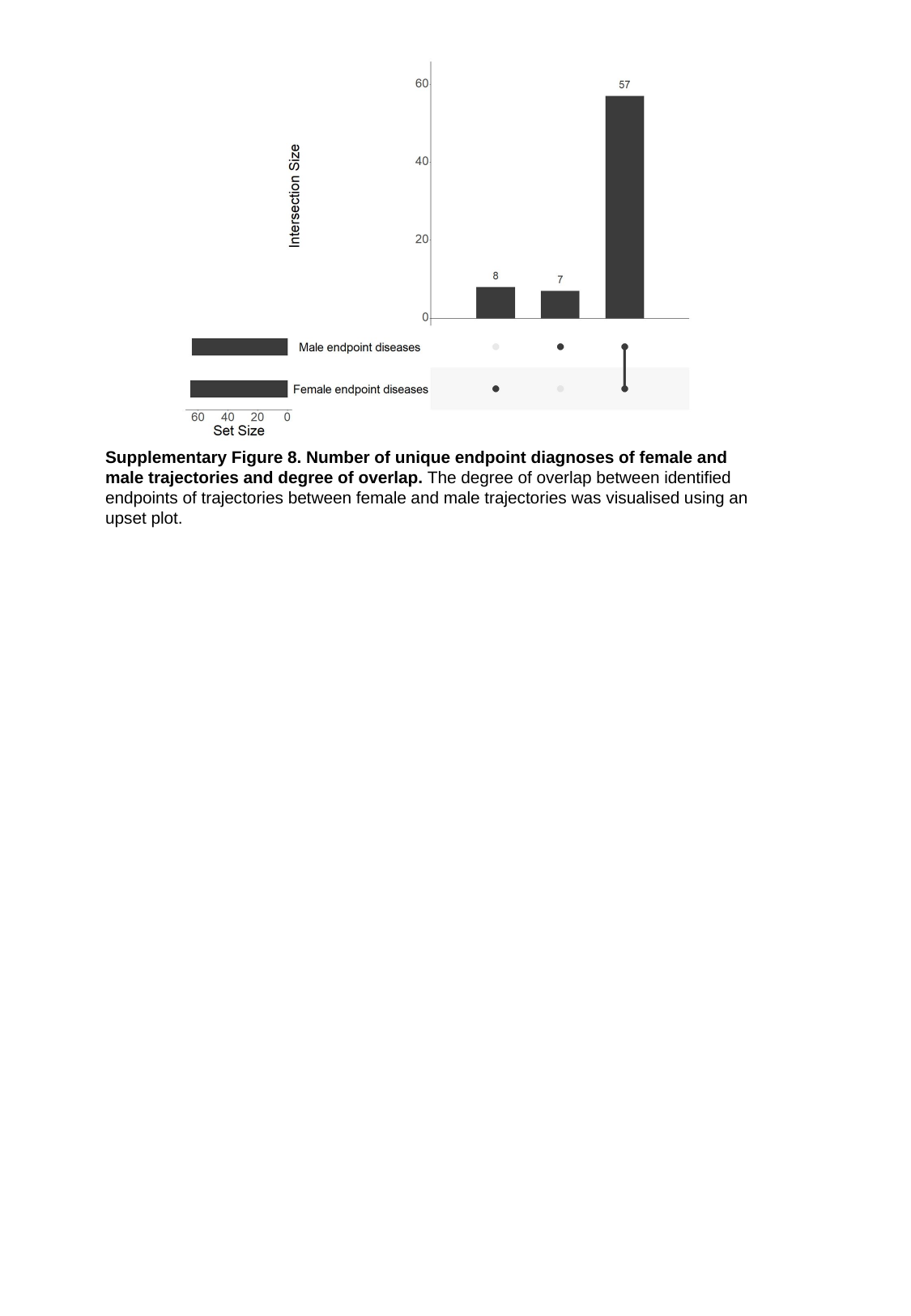

Supplementary Figure 8. Number of unique endpoint diagnoses of female and male trajectories and degree of overlap. The degree of overlap between identified endpoints of trajectories between female and male trajectories was visualised using an upset plot.

## Slide 9
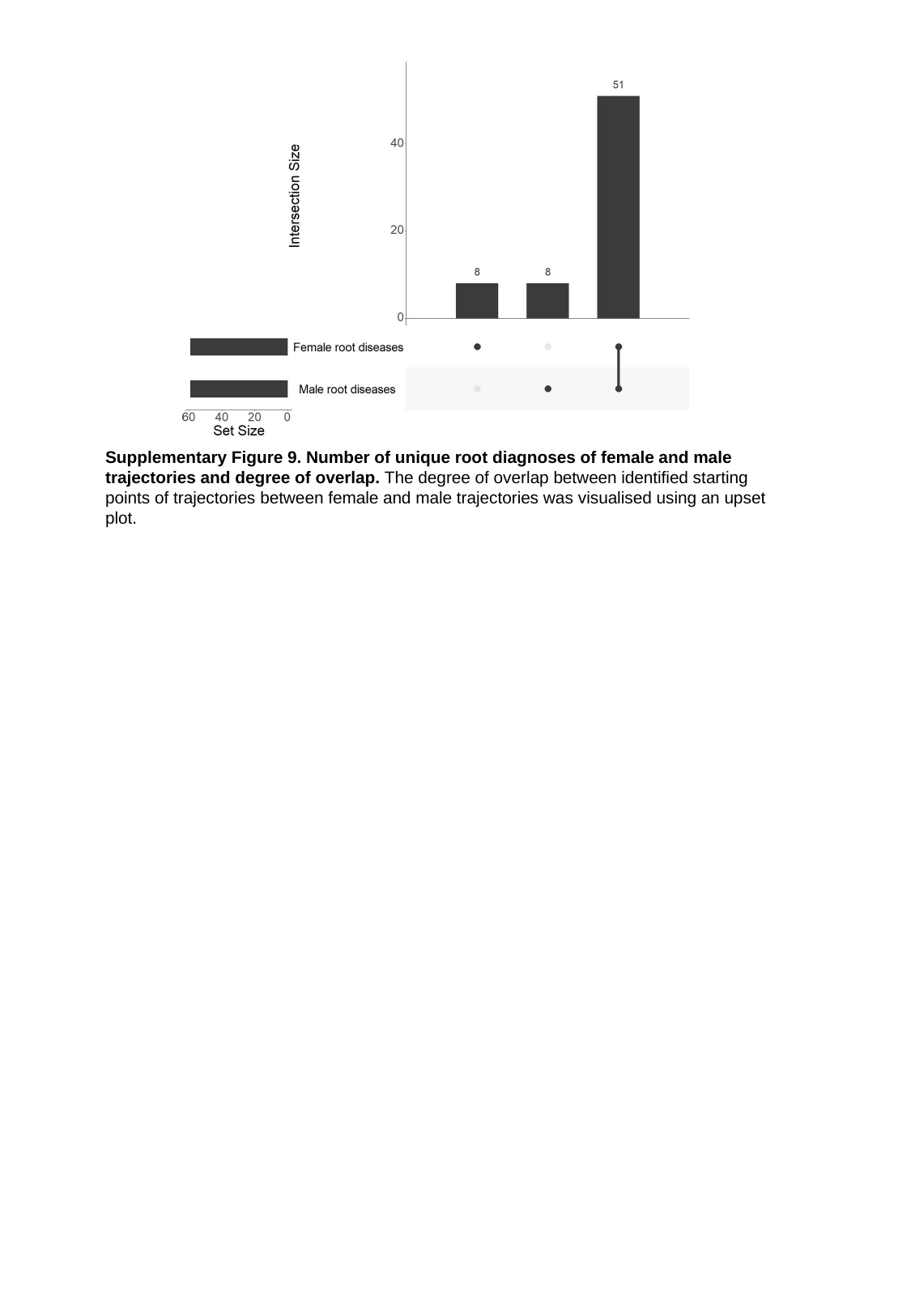

Supplementary Figure 9. Number of unique root diagnoses of female and male trajectories and degree of overlap. The degree of overlap between identified starting points of trajectories between female and male trajectories was visualised using an upset plot.

## Slide 10
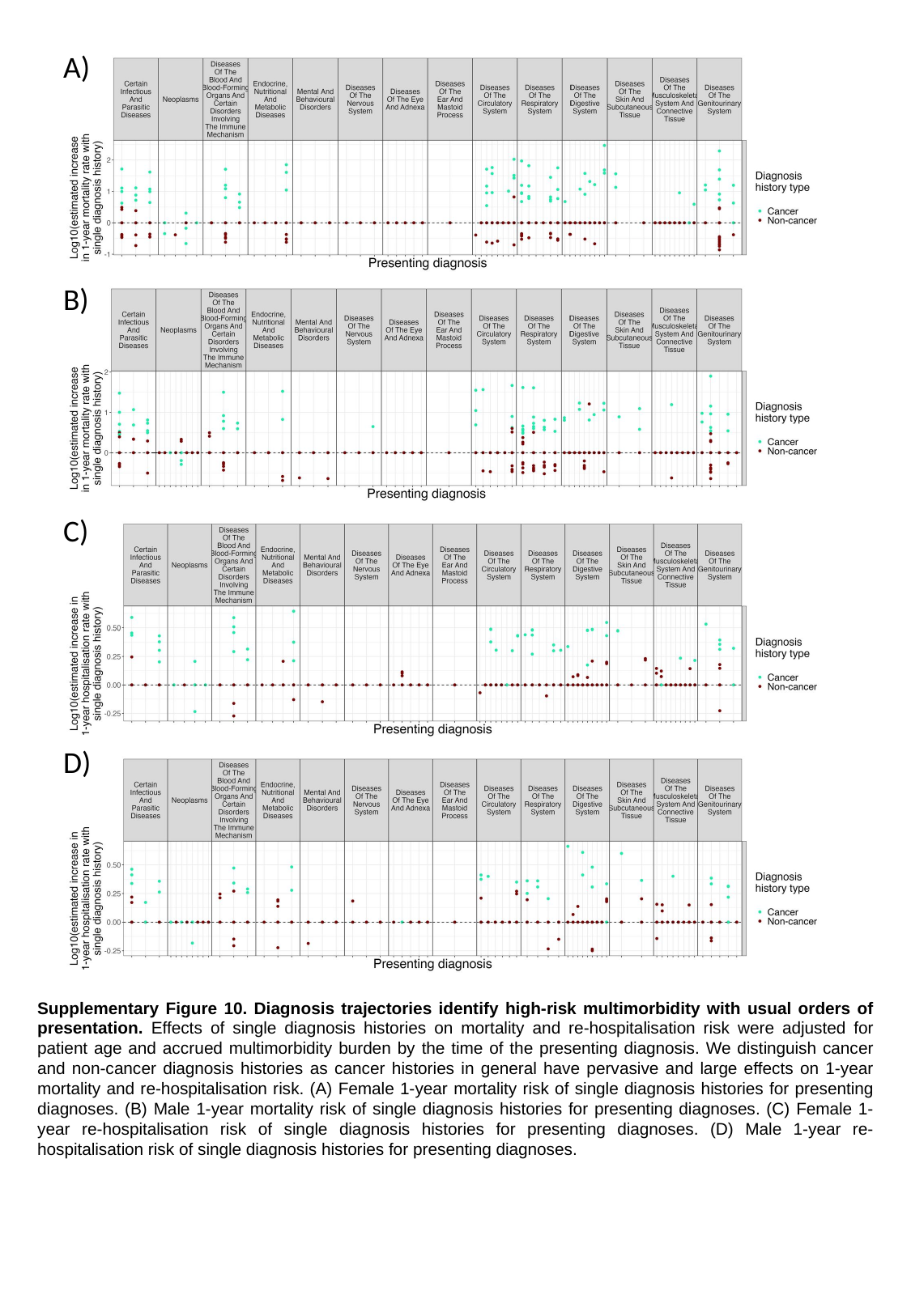

A)
B)
C)
D)
Supplementary Figure 10. Diagnosis trajectories identify high-risk multimorbidity with usual orders of presentation. Effects of single diagnosis histories on mortality and re-hospitalisation risk were adjusted for patient age and accrued multimorbidity burden by the time of the presenting diagnosis. We distinguish cancer and non-cancer diagnosis histories as cancer histories in general have pervasive and large effects on 1-year mortality and re-hospitalisation risk. (A) Female 1-year mortality risk of single diagnosis histories for presenting diagnoses. (B) Male 1-year mortality risk of single diagnosis histories for presenting diagnoses. (C) Female 1-year re-hospitalisation risk of single diagnosis histories for presenting diagnoses. (D) Male 1-year re-hospitalisation risk of single diagnosis histories for presenting diagnoses.

## Slide 11
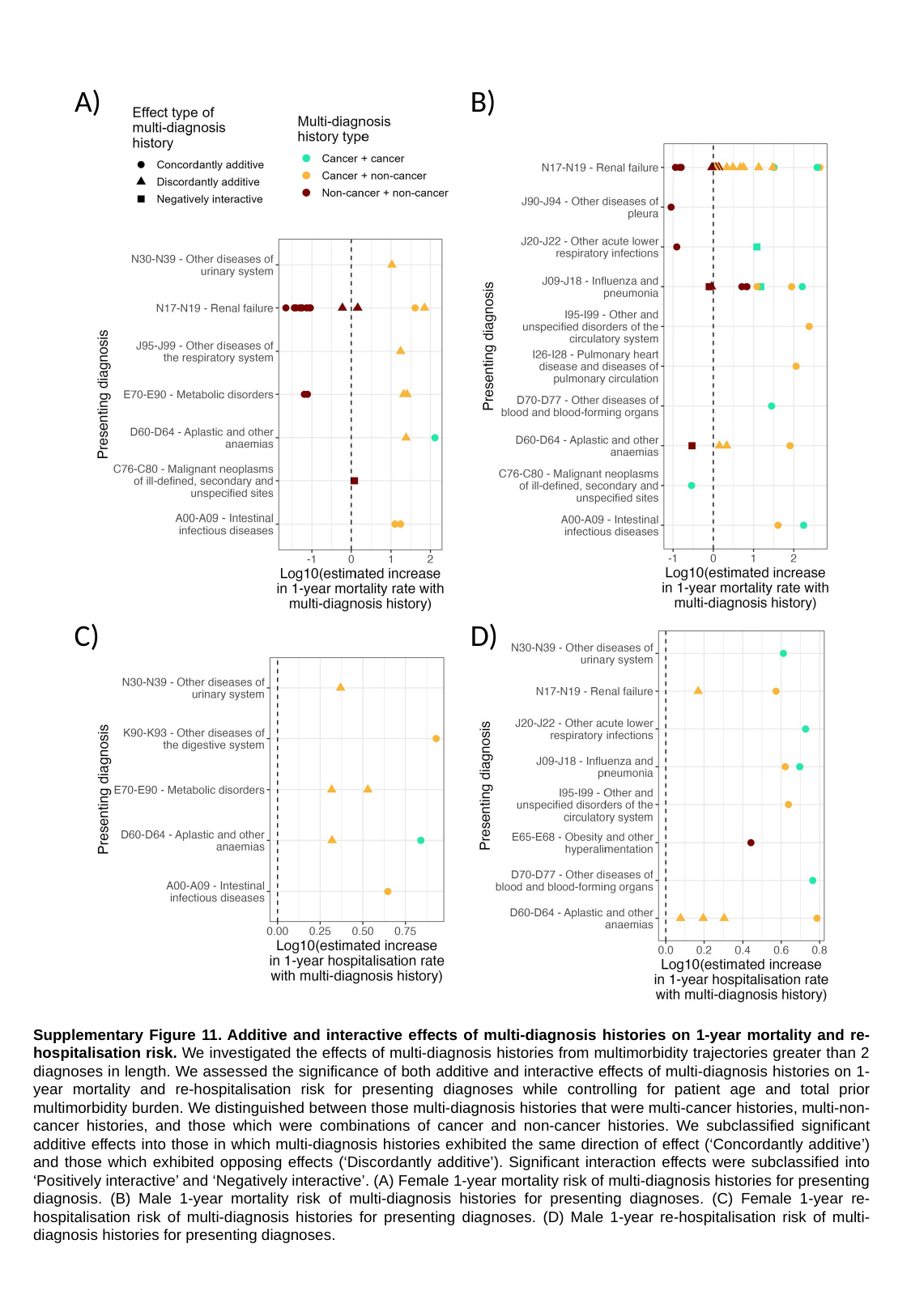

A)
B)
C)
D)
Supplementary Figure 11. Additive and interactive effects of multi-diagnosis histories on 1-year mortality and re-hospitalisation risk. We investigated the effects of multi-diagnosis histories from multimorbidity trajectories greater than 2 diagnoses in length. We assessed the significance of both additive and interactive effects of multi-diagnosis histories on 1-year mortality and re-hospitalisation risk for presenting diagnoses while controlling for patient age and total prior multimorbidity burden. We distinguished between those multi-diagnosis histories that were multi-cancer histories, multi-non-cancer histories, and those which were combinations of cancer and non-cancer histories. We subclassified significant additive effects into those in which multi-diagnosis histories exhibited the same direction of effect (‘Concordantly additive’) and those which exhibited opposing effects (‘Discordantly additive’). Significant interaction effects were subclassified into ‘Positively interactive’ and ‘Negatively interactive’. (A) Female 1-year mortality risk of multi-diagnosis histories for presenting diagnosis. (B) Male 1-year mortality risk of multi-diagnosis histories for presenting diagnoses. (C) Female 1-year re-hospitalisation risk of multi-diagnosis histories for presenting diagnoses. (D) Male 1-year re-hospitalisation risk of multi-diagnosis histories for presenting diagnoses.
